# The applicability of recreation-grade GNSS receiver (GPS watch, Suunto Ambit Peak 3) in a forested and an open area compared to a mapping-grade receiver (Trimble Juno T41)

**DOI:** 10.1371/journal.pone.0231532

**Published:** 2020-04-17

**Authors:** Taeyoon Lee, Pete Bettinger, Chris J. Cieszewski, Alba Rocio Gutierrez Garzon

**Affiliations:** Warnell School of Forestry and Natural Resources, University of Georgia, Athens, Georgia, United States of America; China University of Geosciences, CHINA

## Abstract

Due to developments in global navigation satellite systems (GNSS) and the miniaturization of their components, the usage of Global Positioning System (GPS) is no longer restricted to professional applications, but has become available in various consumer type devices, such as wristwatches. These commercial devices, however, were primarily designed for tracking activities in predominately urban settings and their accuracy has not been tested in forested areas. In this study, we present an assessment of the positional accuracy of a GPS watch (Ambit Peak 3, Suunto, Finland) under different forest cover types, seasons and meteorological conditions within the Whitehall Forest GPS Test Site located in Athens, Georgia, USA. As a standard of comparison, the performance of the GPS watch measurements was juxtaposed to that of a mapping-grade receiver (Juno T41, Trimble Inc., USA). In this study, we analyzed the differences between the determined and control positions using root-mean-square-error (RMSE), along with the distribution of observed positions through the standard deviational ellipse. The results suggest that the seasonal variations contributed to a statistically significant impact on the RMSE values for the GPS watch. However, there were no statistically significant differences in horizontal position accuracy by forest cover-type when using the GPS watch. Furthermore, no significant differences were found in horizontal position accuracy during the leaf-off period between the RMSE values for the GPS watch and those of the mapping-grade receiver. Lastly, the positional accuracies for both types of receivers were found to be weakly, but significantly correlated with fluctuations in air temperature and absolute humidity.

## Introduction

Although some discussions concerning the development of global navigation satellite systems (GNSS) occurred before the 1980s, their notable development began in earnest on the NAVSTAR GPS (United States) and GLONASS (Russia) systems during the 1980s. In the most recent years, there have been further developments by other entities, such as China’s BeiDou/COMPASS and European Union’s GALILEO projects. Each of these four systems provide publicly available signals, which hardware technology manufacturers can subsequently use to develop commercially available GNSS receivers. Consequently, since around the turn of the millennium there has been a rapid development in GNSS software and hardware. These developments have led not only to improvements in the reliability of GNSS, but also to its widespread commercial availability. For instance, the miniaturization of GNSS antennas and chips has allowed them to be incorporated into various types of digital devices, ranging from smartphones or smart wearables to wristwatches [1, 2]. These types of non-traditional GNSS receivers allow people to become accustomed to the use of GNSS technology in their daily lives yet they provide very little information on the quality of data they generate. For instance, while many people may use smartphones to navigate unfamiliar territories, knowledge on the accuracy by which these devices portray horizontal positions on Earth is slowly forthcoming (e.g., [3]). Furthermore, many in the general public are beginning to use GPS watches to navigate or track their activities while exercising, without proper understanding of the impact that various meteorological conditions may have on GPS functionality. Currently, it is uncertain whether the positional information derived from non-traditional types of GNSS receivers can serve as a reliable substitute in non-ideal circumstances, relative to the reliable information collected by traditional mapping-grade, which are often used to acquire sufficient quality positional information demanded by forest management or forestry research professionals [4].

In the forestry literature, GNSS receivers are generally classified into three groups: survey-grade, mapping-grade, and recreation or consumer-grade. In a general sense, the capability of horizontal position accuracy of individual receivers has been positively correlated with their prices [5, 6]. From this perspective, cellular phones and tablets that act as GNSS receivers would fall within the recreation-grade class from both a cost and a horizontal accuracy perspective [7]. Although survey-grade GPS receivers typically provide the highest positional accuracy in forests [8], the cost, knowledge, and skill required to operate these receivers, as well as the residence time required to determine a position (perhaps 20 minutes) hinder their use in typical forestry applications. Concerning mapping-grade receivers, this group typically achieves good static horizontal position accuracy under relatively open sky conditions (sub-meter) and in forests (2–5 meters) [9, 10]. Moreover, due to their moderate price range and the high degree of accuracy that they provide, mapping-grade receivers are commonly used for both forest management and forestry research purposes [4]. On the other hand, recreation-grade receivers generally provide the lowest accuracies, often in the 5–15 m range [11, 12], but are compensated by their low purchase price. This affordability of recreation-grade receivers makes them a relatively accessible and attractive option for non-professional applications. GPS watches can be classified as recreation or consumer-grade receivers because of their price and observed levels of positional accuracy.

As there are few notable brands which produce GPS watches, vigorous debate exists about their accuracy and reliability among the increasing number of people who use them. Since the GPS watch acts as an attractive alternative to a traditional GNSS receiver, its application and limitations should be documented. For instance, GPS watches have been used to monitor children and elderly people in urban areas and to track vehicle speed in motorsports [1, 2]. Although researchers have already attempted to evaluate the usefulness of GPS watches currently offered on the consumer market, the methodology employed has been limited by either addressing the positional accuracy without conducting formal tests, or by conducting tests that failed to include a precise control.

Although some GPS watches have been designed for outdoor recreational purposes, their positional accuracies in forested areas have not yet been evaluated. Similar to urban areas, where artificial structures may interfere with GNSS signals, forested areas are also considered very challenging due to the interference by the topography and canopy cover, which mask and block signals [13–15]. While many studies have examined the accuracy of recreation-grade and mapping-grade GNSS receivers in forested areas [9–11, 16, 17], the horizontal position accuracy of GPS watches in forested areas has yet to be evaluated. Furthermore, while a few studies have investigated the relationship between meteorological conditions and recreation-grade and mapping-grade GNSS receiver accuracy, no study to date has investigated the relationship between meteorological conditions and the accuracy of a GPS watch [11, 16–18].

Therefore, this study was conducted to evaluate the static horizontal position accuracy of a GPS watch when used under different cover types, seasons, and meteorological conditions. The performance of the GPS watch was also compared to that of a mapping-grade GNSS receiver under identical environmental conditions. The hypotheses of this research are as such:

H1: The static horizontal position accuracy of the GPS watch is not significantly different between the leaf-on and the leaf-off seasons.

H2: The static horizontal position accuracy of the GPS watch is not significantly different between its uses in a coniferous forest, deciduous forest, and an open field.

H3: The static horizontal position accuracy of the GPS watch does not significantly change with variations in meteorological conditions (air temperature, relative/absolute humidity, atmospheric pressure, and wind speed).

H4: The static horizontal position accuracy of the GPS watch does not significantly differ from that of a mapping-grade GNSS receiver.

## Materials and methods

We conducted this study to assess the quality of static horizontal position data as measured by two GNSS receivers: a Suunto GPS watch (Ambit Peak 3, Suunto, Finland) and a Trimble GNSS device (Juno T41, Trimble Inc., USA). The Suunto GPS watch is much lighter than most other GNSS receivers, weighing approximately 89 grams. The watch is equipped with the GPS L1 patch antenna (15×15×4 mm) made by Patron Co. Ltd (Korea), which is relatively small and contained inside the watch. Furthermore, the watch contains a rechargeable battery, which can last up to 200 hours depending on the amount and type of activity. For static horizontal data collection, the display of the GPS coordinate system and compass declination was set to UTM NAD1983 and as 5.5° degrees west, respectively. Although the watch allows users to change the GPS fixed intervals with three different levels, namely a “Best” interval (~ 1 sec), a “Good” interval (~ 5 sec), and an “OK” interval (~ 60 sec), this is only possible when it is tracking activities (dynamic horizontal data collection). Unlike many higher-grade receivers, the Suunto GPS watch did not allow for defining masks for the maximum positional dilution of precision (PDOP) and the minimum signal-to-noise ratio (SNR, the ratio between received power of signal and noise power).

The Trimble GNSS unit (Juno T41, Trimble Inc., USA) is considered a mapping-grade GNSS receiver with a wide range of operating temperatures (-30°C to 60°C). It is equipped with a multi-function antenna (GPSGLONASS08N-S3-03-A, Inpaq technology Co., Ltd., Taiwan) that is larger than that of the GPS watch (70×43.18×9 mm). SOLO Forest software (Trimble Forestry Automation 2009) is used to collect and save the data. The maximum PDOP level on this receiver was set to 8, a moderate setting for the satellite geometric arrangement, and the minimum SNR was set to 4. The real-time, space-based augmentation was enabled using the wide area augmentation system (WAAS) satellite signal. Although the Suunto GPS watch and Trimble GNSS receiver are fundamentally different, a comparison between their accuracies would be helpful in ascertaining the current utility and limitations of recreation-grade GNSS receivers.

To assess the horizontal position accuracy of the two GNSS receivers under forested conditions, we conducted this study at the Whitehall Forest GPS Test Site (gps-test-site.uga.edu) located in Athens, Georgia (USA). This test site was established in 2004 by professional surveyors, and it contains 3 monuments and 37 control points. The three monuments consist of an aluminum pin, about 9 cm in diameter and 1 m long, driven into the ground so that the top is flush with the ground level. The 37 control points each consist of a brass survey cap attached to a piece of rebar (about 0.5 m long), encased in cement (about 30 cm in diameter), where the survey cap is flush with the ground level. The positions of the three survey monuments were established using an Ashtech Locus survey-grade GNSS receiver. Data were collected for these three survey monuments over the span of four hours and subsequently processed using the Online Positioning User Service (OPUS), which is managed by the U.S. Department of Commerce, National Oceanic, and Atmospheric Administration (www.ngs.noaa.gov/OPUS). These three monuments have been accepted into the National Spatial Reference System (NSRS), with their positional precision certified to under 2 cm. Using the three monuments as a base, professional surveyors conducted a closed traverse survey of the 37 control points using a Topcon GTS-211D instrument. The resulting closure of the closed traverse network was estimated to be 1/92,137, and the positions of the 37 control points are estimated to have similar horizontal position accuracy of approximately 2 cm. It is considered, therefore, that the Whitehall Forest GNSS Test Site is a highly accurate model around which GNSS equipment could be tested.

**Fig 1:**
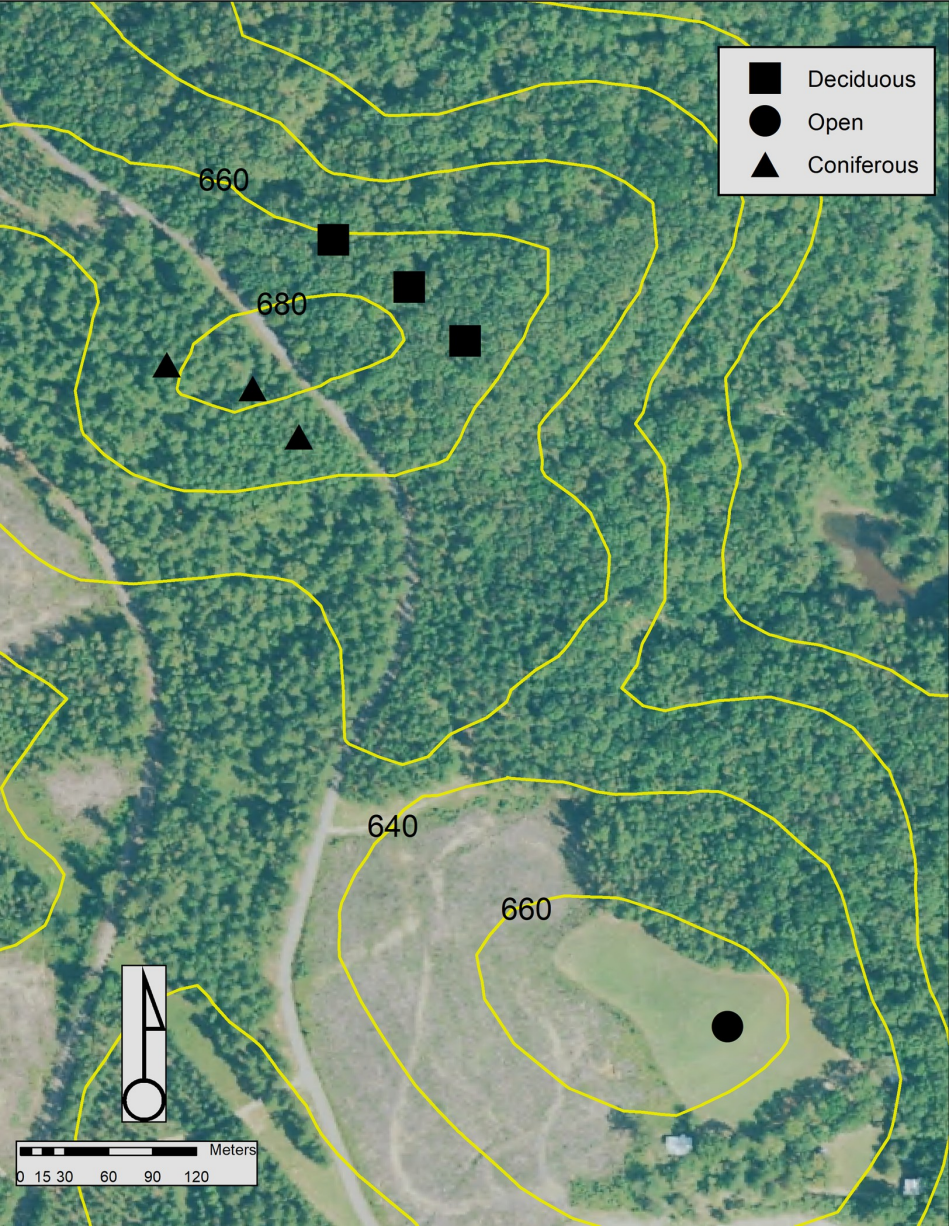
The surveyed area with control points, located at the Whitehall Forest GPS Test Site in Athens, Georgia (USA).

Three of the control points that we selected for this study were located within an older coniferous forest, containing *Pinus echinata* and *Pinus taeda* which were 70 to 80 years age. The density of this area was estimated to be 223 trees ha^-1^, and the basal area was estimated to be 26.0 m^2^ ha^-1^. Three other control points we selected were located within an older deciduous forest consisting of *Quercus* spp., *Carya* spp., *Ostrya virginiana*, and other species, also 70 to 80 years age. The density in this area was estimated to be 126 trees ha^-1^, and the basal area was estimated to be 23.0 m^2^ ha^-1^ (Fig 1). These six control points were visited 17 times between 3:00 p.m. and 4:30 p.m. during the leaf-on season (September-October) and 17 times during the leaf-off season (December). In total, this resulted in 51 independent visits to control points in the coniferous forest and 51 independent visits to control points in the deciduous forest during each season. Furthermore, an open field NSRS monument was visited 17 times during each season.

When the GNSS data were collected, we randomized the forest cover type (coniferous or deciduous), and further randomized the order of the three control points within each forest cover type, in an attempt to avoid potential biases. During each visit to a control point, each of the two GNSS receivers was positioned on top of a monopod (1.3 m) equipped with a leveling device. Researchers consistently positioned themselves on the north side of control points as the data were collected. Furthermore, an effort was made to ensure that the internal GNSS antenna of each device was positioned directly above the control points as the data were collected. The open field measurement was visited after the coniferous and deciduous control points were surveyed since it was about 1 km away by road. The Trimble GNSS receiver was allowed to warm-up (approximately 5 min) before its use. The Suunto GPS watch was always on and was therefore assumed to be ready for data collection purposes. The Suunto GPS watch collected a single waypoint at each control point during all visits. On the other hand, at each particular visit, about 15 position fixes per point were collected at 1-second intervals using the Trimble receiver. These position fixes were subsequently averaged, prior to downloading the data from the Trimble device, to produce a single position fix during each visit to each control point. We followed this protocol to be consistent with normal field data collection practices of foresters. Since the data collected by the Suunto GPS watch was saved in degrees (longitude and latitude to the specificity of the sixth decimal place) using the WGS 1984 coordinate system, this data were converted to UTM coordinates using ArcMap GIS software (version 10.6.1, Esri Inc., Redlands, CA, USA) for further processing. The Trimble GNSS device saved data in both formats of WGS and UTM coordinates. The difference (static horizontal position error) between each determined position and the associated control point position was computed using the root mean square error (RMSE), as has been done in many previous studies (i.e., Bettinger and Fei 2010, Bettinger and Merry 2012, Danskin et al. 2009, Sigrist et al. 1999, Weaver et al. 2015). RMSE can be calculated as follows:

$$\text{RMSE}=\sqrt{\sum_{i}^{n}\left({\left(x_{i}-x\right)}^{2}+{\left(y_{i}-y\right)}^{2}\right)/n}$$

Where *n* is the total number of observations in a visit; *i* is the *i*^th^ observation of the visit; *x*_*i*_ and *y*_*i*_ are respectively the easting and northing of the *i*^th^ observations; and *x* and *y* are the true easting and northing of the associated control point. Unlike the standard deviation which assesses accuracy using deviations from a mean value, the RMSE was considered as a good estimator to evaluate its accuracy because it represents the deviation from the truth, not the from the mean [19]. In addition to the RMSE, the root squared error of the mean (RSEM) is calculated based on the mean center’s coordinates as follows:

$$\text{RSEM}=\sqrt{{\left(\frac{\sum_{i}^{n}(x_{i}-x)}{n}\right)}^{2}+{\left(\frac{\sum_{i}^{n}(y_{i}-y)}{n}\right)}^{2}}$$

Where *n* is the total number of observations in a visit; *i* is the *i*^th^ observation of the visit; *x*_*i*_ and *y*_*i*_ are the longitude and latitude of the *i*^th^ observations; and *x* and *y* are the true easting and northing of the associated control point. Standard deviational ellipses were also calculated to investigate the trends of determined positions, to assess whether these are related to cover type or season. Among standard deviational ellipses parameters, the anisotropic ratio was calculated as follows:

$$I_{a}=\frac{R-r}{R}\times 100\%$$

Where *I*_*a*_ is anisotropic ratio; *R* and *r* are the length of ellipse long and short axis, respectively.

Meteorological data, including air temperature, relative/absolute humidity, wind speed and barometric atmospheric pressure, were obtained for the relevant time period of each visit from a local weather station (Athens, Georgia airport). This data was used in statistical tests to determine their correlation with horizontal position error. In particular, these meteorological variables were selected due to their potential influence on GNSS signals as they pass through Earth's lower troposphere. The aforementioned local weather station reported these metrics in one-hour intervals, and thus a linear interpolation was performed to estimate their values at approximately the time of data collection. The absolute humidity, on the other hand, was calculated using a web-based calculator (https://planetcalc.com/2167/) because it was not directly monitored. Since the majority of RMSE values were normally distributed, we applied the one-way ANOVA, Kruskal-Wallis test, the Student’s *t*-test and the Welch’s *t*-test to test the hypotheses noted above using R Studio software (version 1.1.463, RStudio, Inc., Boston, MA, USA). The RSEM and parameters of standard deviational ellipses were calculated with ArcMap GIS software (version 10.6.1, Esri Inc., Redlands, CA, USA).

## Results

**Fig 2:**
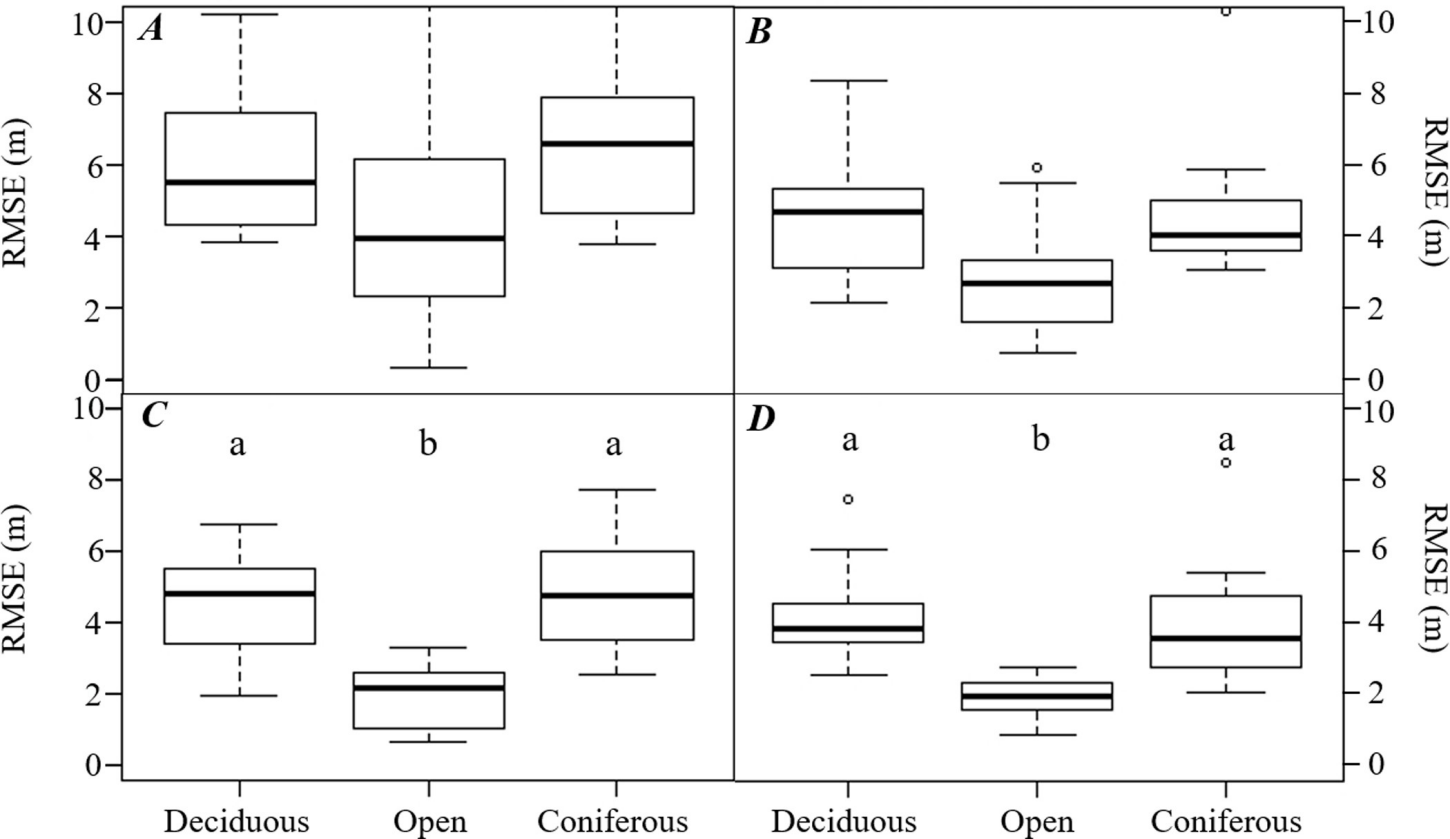
Boxplots for horizontal positional accuracy (RMSE) by equipment type (GPS watch: *A*, *B*; Mapping-grade GNSS receiver: *C*, *D*), cover type, and season (Leaf-on: *A*, *C*; Leaf-off: *B*, *D*). *Different letters* indicate significant differences (For *A* and *B*, Scheffe’s Test at *p-value* < 0.05; for *C* and *D*, Dunn’s Test at *p*-value < 0.05).

Concerning H1, the observed RMSE values were analyzed using a Welch’s *t*-test according to the season in which the data were collected. We found a significant difference in the mean RMSE values between the leaf-on and leaf-off seasons when using the GPS watch (*p-value* < 0.001) (Fig 2). For the Trimble GNSS receiver, meanwhile, no statistically significant differences were observed in the static horizontal position accuracy for either the leaf-off (mean = 3.50 m, standard deviation = 1.85 m) or leaf-on seasons (mean = 4.03 m, standard deviation = 2.34 m) (Student’s *t*-test, *p-value* = 0.21). Therefore, we reject H1 and conclude that horizontal position accuracy of the GPS watch was significantly different between two seasons.

**Fig 3:**
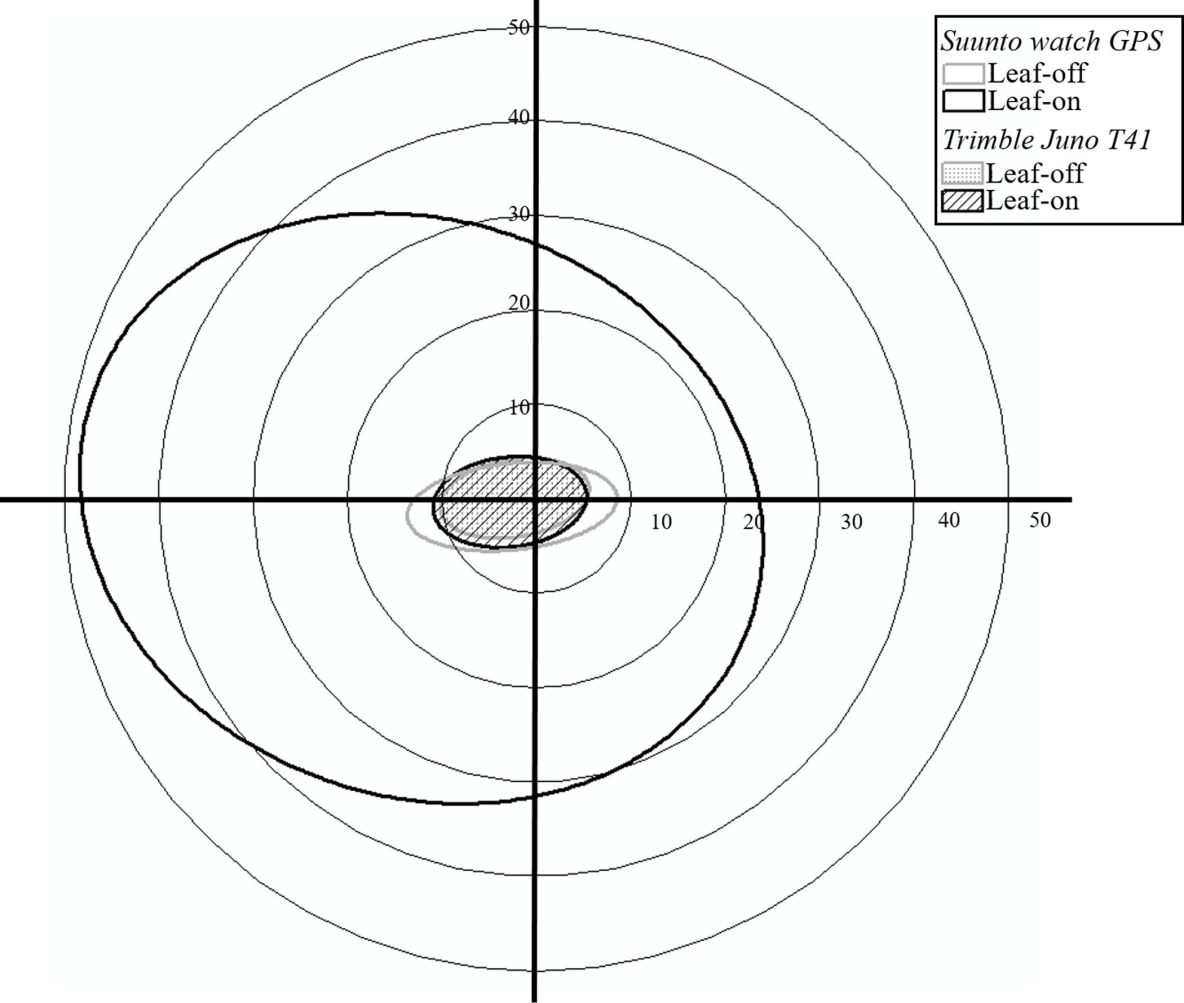
The distributions and standard deviational ellipses of translocated GPS points data obtained by different types of equipment during different seasons.

To represent how the determined GPS positions were spread around the control points, we used the standard deviational ellipse method for point data distribution analysis (Fig 3). The orientation angle of the GPS watch during the leaf-on season was 109.54°, and its *I*_*a*_ was 17.27% (Table 1). This small *I*_*a*_ value suggests that the GPS points might have been distributed in a circular spread rather than an elliptical distribution (Table 1). When considered in conjunction with the area of an ellipse, the observed GPS data points for the GPS watch during the leaf-on season were widely spread from the center of the standard deviational ellipse in a circular distribution area. In contrast, during the leaf-off season, the orientation angle of the GPS watch was 84.17°, and *I*_*a*_ was 59.07% (Table 1). The ascertained area of ellipse during the leaf-off season was smaller than the area during the leaf-on season. This indicates that the observed data points collected during the leaf-off season were more clustered and distributed more closely in relation to the control points.

**Table 1:** The summary of elliptical parameters measured by different GPS equipment during different seasons (RSEM = root squared error of the mean; Mean X coordinate = the mean of difference between observed X coordinates and control X coordinate; Mean Y coordinate = the mean of difference between observed Y coordinates and control Y coordinate; *I*_*a*_ = the anisotropic ratio).

Metrics Device	Leaf-on time period				Leaf-off time period			
	All three sites	Coniferous forest	Deciduous forest	Open field	All three sites	Coniferous forest	Deciduous forest	Open field
*Suunto watch GPS*								
RSEM (m)	12.21	10.06	15.81	15.01	2.70	3.11	2.20	3.94
Mean X coordinate (m)	-12.16	-9.17	-14.40	-14.43	-2.56	-2.77	-1.95	-3.72
Mean Y coordinate (m)	-1.04	4.13	-6.51	4.13	-0.85	-1.41	-1.01	1.29
Angle of rotation (°)	109.54	122.54	48.06	112.77	84.17	84.19	149.54	82.45
*I*_*a*_ (%)	17.27	37.61	18.04	65.35	59.07	63.01	12.66	83.95
Area of ellipse (m^2^)	3525.6	2610.05	3881.4	2398.05	160.37	187.46	56.24	169.13
*Trimble Juno T41*								
RSEM (m)	2.82	2.93	2.49	1.65	2.27	2.39	1.72	1.69
Mean X coordinate (m)	-2.79	-2.85	-2.42	-0.65	-2.27	-2.39	-3.72	-1.20
Mean Y coordinate (m)	-0.35	-0.67	-0.57	1.52	0.06	0.13	1.29	1.19
Angle of rotation (°)	83.82	133.72	31.25	161.87	81.06	52.80	30.82	32.27
*I*_*a*_ (%)	41.78	19.96	18.26	38.16	48.20	4.46	20.61	21.50
Area of ellipse (m^2^)	122.33	77.69	62.37	4.47	102.85	54.40	51.92	2.84

The point data distribution analysis for the Trimble receiver did not show any differences in response to the season. For this analysis, we obtained an orientation angle of 83.82°, and an *I*_*a*_ of 41.78% during the leaf-on season (Table 1). Similarly, the distribution of observed GPS points during the leaf-off season had an orientation angle of 81.06° and an *I*_*a*_ of 48.20%. Here, the *I*_*a*_ values were moderately substantial, suggesting that the GPS points were comparatively spread out on the longest axis of the ellipse (Fig 3). In both seasons, the areas of the ellipses were very similar to each other, and the centers of the ellipses were very close to the control point in every case.

**Fig 4:**
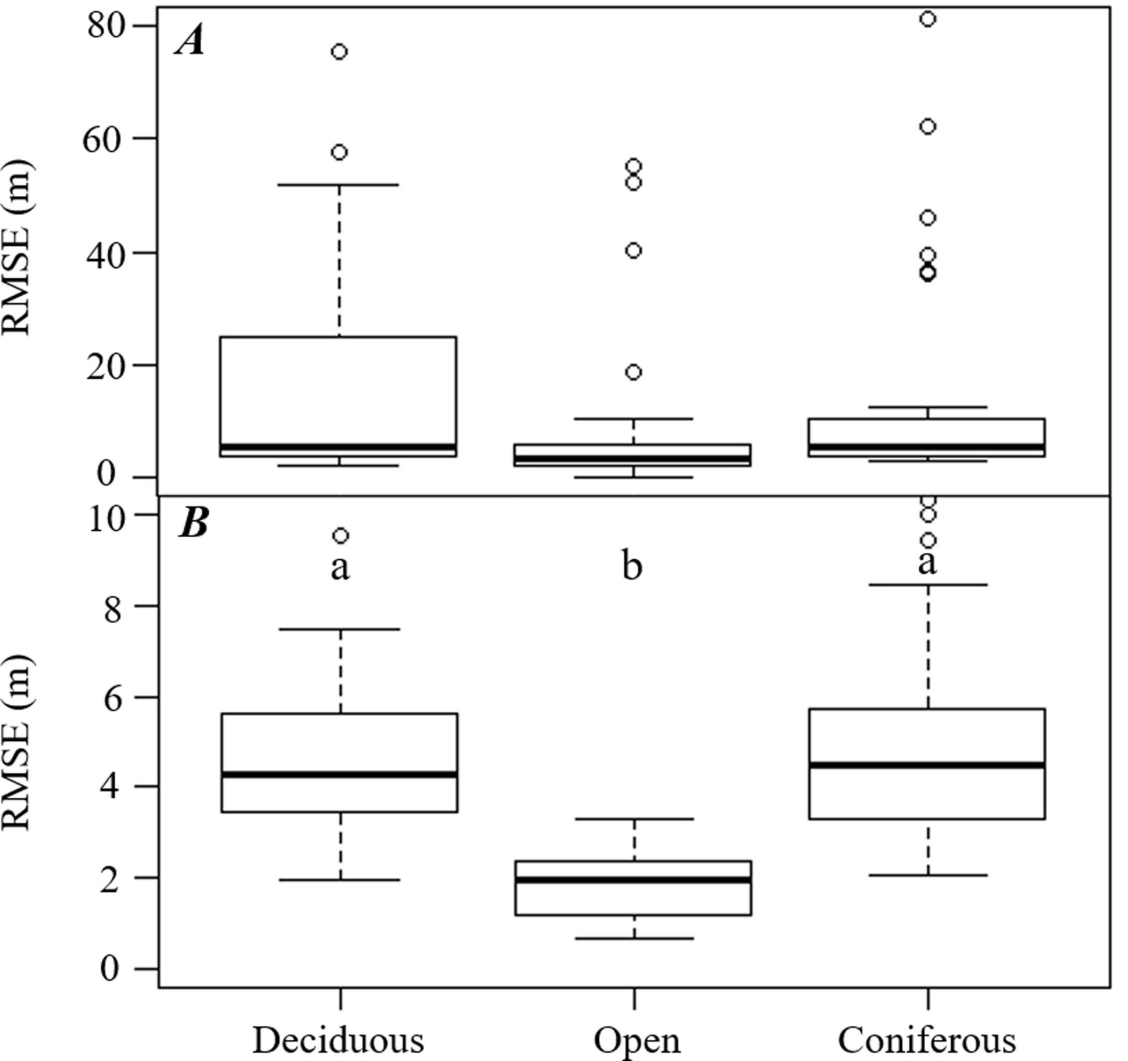
Boxplots describing horizontal positional accuracy (RMSE) by equipment type (GPS watch: *A*; Mapping-grade GNSS receiver: *B*) and cover type. *Different letters* indicate significant differences (Dunn’s Test at *p-value* < 0.05).

Regarding H2, the RMSE values observed by GPS watch and Trimble receiver were analyzed using a one-way ANOVA and Kruskal-Wallis test, to determine whether cover type affected each instruments’ horizontal position accuracy. It was found that there was no statistically significant difference between cover types using the GPS watch, as shown in the results (*F*_*2*,*99*_ *=* 0.242, *p-value* = 0.786) (Fig 4). Although the minimum (best) RMSE was observed in the open field (Table 2), there was no statistically significant difference in the response variable to the cover types in either the leaf-on season (*F*_*2*,*48*_ *=* 0.485, *p-value* = 0.619) or the leaf-off season (*F*_*2*,*48*_ *=* 0.224, *p-value* = 0.8). However, we found statistically significant differences in the accuracy of the Trimble GNSS receiver in the cover types regardless of the season (*p-value* < 0.01) (Fig 4). The lowest RMSE values were found in the open field, and this was significantly different compared to the RMSE values from the forested cover types. There was no significant difference in RMSE values observed in the deciduous and coniferous forest areas. In sum, for the GPS watch, we could not reject H2; however, for the Trimble GNSS receiver, we reject H2, as we found that static horizontal position accuracy varies significantly depending on the cover type.

**Table 2:** Summary results of dataset, with outliers, of the horizontal position error of the GPS watch and mapping-grade GNSS receiver at the Whitehall Forest GPS Test Site in Athens, Georgia (USA).

Metrics Device	Leaf-on time period				Leaf-off time period			
	All three sites	Coniferous forest	Deciduous forest	Open field	All three sites	Coniferous forest	Deciduous forest	Open field
*Suunto watch GPS*								
Mean RMSE (m)	24.25	22.06	29.63	9.27	5.62	6.47	4.52	3.32
Minimum RMSE (m)	0.32	3.80	3.83	0.32	0.77	3.06	2.18	0.77
Maximum RMSE (m)	105.80	81.17	85.32	105.80	55.06	36.61	8.36	55.06
Standard deviation of RMSE (m)	27.48	23.72	25.91	32.86	8.59	7.95	1.63	12.76
*n*	119	51	51	17	119	51	51	17
*Trimble Juno T41*								
Mean RMSE (m)	4.03	5.40	4.81	1.87	3.50	4.33	4.29	1.88
Minimum RMSE (m)	0.66	2.58	1.96	0.66	0.89	2.05	2.53	0.89
Maximum RMSE (m)	10.33	10.33	9.56	3.29	10.00	10.00	7.46	2.77
Standard deviation of RMSE (m)	2.34	2.25	1.89	0.92	1.85	2.14	1.29	0.54
*n*	119	51	51	17	119	51	51	17

The RSEM values for the GPS watch were much higher during the leaf-on season than during the leaf-off season. During the leaf-on season, the highest and lowest RSEM values were found in the deciduous forest and coniferous forest, respectively (Table 1). Otherwise, during the leaf-off season, the highest and lowest RSEM values were obtained in the open field and deciduous forest, respectively. The mean *X* coordinate values were negative regardless of the season and cover type, indicating that the observed points were consistently located to the west of the control point (Table 1). Furthermore, the areas of the ellipses during the leaf-on season were much larger than during the leaf-off season. Meanwhile, the RSEM values for the Trimble receiver were found to be similar to the RSEM values for GPS watch during the leaf-off season. The highest and lowest RSEM values were found in the coniferous forest and open field respectively, regardless of the season. The mean *X* coordinate values were also negative regardless of season and cover type, and the areas of the ellipse were also similar to the those of the GPS watch during the leaf-off season.

For H3, the correlation coefficient *r* values were summarized in Table 3. These values indicate that air temperature and absolute humidity were significantly correlated with RMSE, regardless of equipment type. For both the Suunto watch and the Trimble receiver, weak positive correlations were found between the RMSE and the air temperature (0.26 and 0.20 for GPS watch and Trimble receiver, respectively) and between the RMSE and the absolute humidity (0.22 and 0.15 for GPS watch and Trimble receiver, respectively). Except for the temperature and absolute humidity, there were no other observed significant correlations between the horizontal position accuracy and meteorological factors. In sum, we only found weak positive correlation between RMSE values and air temperature or absolute humidity.

**Table 3:** Summary of Pearson correlation coefficients for RMSE and meteorological factors.

Env. factors Device	Air temp. (°C)		Relative humidity (%)		Absolute humidity (g⸱m^-3^)		Wind speed (mph)		Barometric pressure (inches)	
	r	*p -value*	*r*	*p-value*	*r*	*p -value*	R	*p -value*	*r*	*p-value*
*Suunto Watch GPS*	0.26 ***	< 0.001	-0.07	0.2997	0.22 ***	< 0.001	-0.08	0.208	0.02	0.7984
*Trimble Juno T41*	0.20 **	0.002	-0.08	0.2246	0.15*	0.02	-0.02	0.801	0.05	0.4855

*** Statistically significant difference, *p-value* < 0.001.

** Statistically significant difference, *p-value* < 0.01.

* Statistically significant difference, *p-value* < 0.05.

Finally, concerning H4, we applied the Welch’s *t*-test to compare RMSE values observed by the GPS watch and the mapping-grade GNSS receiver, in response to the cover type and seasons. It was found that there were no significant differences observed during the leaf-off season regardless of the cover type (Table 4). Otherwise, there were significant differences during the leaf-on season, and across both seasons. In conclusion, we partially fail to reject H4 because there was no significant difference in RMSE values by GPS watch and mapping-grade GNSS receiver during the leaf-off season. However, we reject H4 with respect to the leaf-on season.

**Table 4:** Summary of the comparison between RMSE observed by different equipment type using a Welch’s *t*-test (*p-value* < 0.05).

Forest cover type Period	Coniferous Forest	Deciduous Forest	Open field
	*p -value*	*p -value*	*p -value*
Whole year	*p* = 0.007*	*p* = 0.002*	*p* = 0.013*
Leaf-on	*p* = 0.011*	*p* = 0.001*	*p* = 0.029*
Leaf-off	*p* = 0.297	*p* = 0.648	*p* = 0.215

* Statistically significant difference, *p-value* < 0.05.

## Discussion

Using a Trimble mapping-grade receiver as a basis of comparison, this study attempted to assess the accuracy of a recreation-grade GPS watch in response to the seasonal fluctuations and variations in the canopy cover and to investigate the correlation between the accuracy of the watch and meteorological factors that included humidity, air temperature, atmospheric pressure, and wind speed. The accuracy of the GPS watch was found to be significantly enhanced during the leaf-off season relative to the leaf-on season. In comparison, the accuracy of mapping-grade GNSS receiver was found to not be affected by seasonal fluctuations. One study [5] also had similar results, in that the significant effect in response to the change in season was only found when it was measured using the recreation-grade GNSS receiver, not with the mapping-grade GNSS receiver. Another study [17] conducted at the same site also found that the static horizontal accuracy was not affected by season using a mapping-grade GNSS receiver. Cumulatively, these results indicated that mapping-grade GNSS receivers might not be affected by the seasonal fluctuation, whereas the recreation-grade GNSS receivers do appear to be more sensitive to the seasonal fluctuation. Although vegetation obstruction introduces a degree of error in the forested area through the blockage of satellite signals or through multipath signals [11, 20, 21], the defoliation during the winter season in the deciduous forests had a weak, but positive effect on enhancing the static horizontal position accuracy. Indeed, there was a significant increase in accuracy observed in every cover type (coniferous forest, deciduous forest, and open field) during the leaf-off season in this study. In other studies, the improvement in accuracy during leaf-off season in the deciduous forest was not observed [5, 11, 17]. One possible explanation for the improvement of GPS watch accuracy during the leaf-off season, regardless of cover type, is the cumulative error caused by different meteorological conditions such as relatively low temperature and absolute humidity during leaf-off season that had a positive correlation with the RMSE values (negative correlation with accuracy) [16, 19, 22, 23]. Furthermore, due to the improvements in accuracy observed during leaf-off seasons, there was no significant difference between accuracy of GPS watch and mapping-grade receiver during this season. This suggests that the GPS watch could be used to replace the mapping-grade receivers when considering cost efficiency, especially during leaf-off season.

Even though the outliers were also considered in this study, the static horizontal positions determined by the recreation-grade GNSS receiver and the mapping-grade GNSS receiver in the forested conditions had relatively low mean RMSE values in the range of 4.52 to 29.63 m and 4.29 to 5.40 m, respectively. These results were similar to other studies’ results (recreation-grade GNSS receiver: 7 to 12 m, and mapping-grade GNSS receiver: 1 to 5 m) that were conducted at the same location [9, 11, 12, 17]. These results confirm that our research was conducted reliably and indicate that the watch-type recreation-grade GNSS receiver maintained similar degree of accuracy to the conventional handheld type of recreation-grade GNSS receiver in a forested area. The significant effects cover type had on static horizontal position accuracy were only observed when the mapping-grade GNSS receiver was used, not when the GPS watch was used due to the large variances observed. However, this significant difference was only found between the open field and forested area (coniferous and deciduous forest), not between the coniferous and deciduous forests. These results contrasted with other studies that have shown a significant difference in static horizontal position accuracy when using a recreation-grade GNSS receiver among varying forest cover type [5, 11, 12, 24]. However, other studies [16, 17] did not find a significant difference in static horizontal position accuracy between forest cover types; yet, these studies employed mapping-grade GNSS receivers. These results indicate that the cover type itself is not the main factor affecting the static horizontal position accuracy. Rather, it suggests that canopy coverage might be the primary consideration when determining the GPS receiver accuracy [19]. Indeed, the GPS accuracy was shown to be improved in a young coniferous forest relative to an old coniferous forest [11, 24, 25] and further accuracy improved in post-thinning conditions compared to the pre-thinning conditions [9].

We applied the standard deviational ellipse to investigate the direction or tendency of error, depending on the season and cover type; this study was the first of its type in forestry research to use GNSS data in this manner. As the standard deviational ellipse is a measure of the distribution of observed points, it provided information about the data concentration, including orientation (determined by the direction of the longest axis of observed points), anisotropic ratio (ratio of the longest and shortest axes), and the area of the ellipse intuitively through images and quantification [26]. Although the area of ellipse was considerably smaller during leaf-off season than during the leaf-on season regardless of GPS receiver type, a specific tendency in the orientation and the area of an ellipse was not observed in response to the cover type. However, the mean center of each ellipse was located on the western side of the control points regardless of the GPS receiver types. There have been a few studies considering the direction of the error [9, 11, 18]. One study [9] using evaluations of rose diagrams confirmed that the bias in error changes in response to thinning. Our results showed that there were more points distributed on the western side of the control points as opposed to the eastern side. Although the location of nearby trees around control points was not investigated in our study, Bettinger and Merry [18] suggested that the vegetation near the control point influenced the direction and magnitude of the positional error. Another possible explanation for the biased distributions of the observed points is the movement of satellites within the constellation or the Earth’s rotation. Finally, while we used the average value of 15 position fixes from the Trimble device to evaluate these, the use of the original 15 position fixes may have revealed some balanced error. However, at this time we are unable to determine whether the standard deviational ellipses would have been different had we evaluated them in this manner.

Prior studies have indicated that there was no correlation between meteorological variables of the lower troposphere and static horizontal position accuracy [11, 16–18]. The only study [16], among the aforementioned studies, found a significant and negative correlation between air temperature and RMSE values at the deciduous forest using a mapping-grade GNSS receiver. In our study, however, we found a significant, but weakly positive correlation between air temperature and RMSE regardless of GPS receiver type. The air temperature appears to have receiver-specific effects on horizontal position accuracy, as there was no consistent correlation between air temperature and RMSE values in other studies, regardless of GPS receiver types. Other studies have considered the correlation between relative humidity and RMSE [11, 16–18], but this study investigated the correlation between absolute humidity and RMSE, in which it found a significant, albeit weakly positive correlation. This phenomenon could be explained by the influence atmospheric water vapor has upon the travel of the GPS signal from the satellite. This could be achieved either by delaying the GPS signal propagation, thereby reducing the signal speed, or by causing additional multipath effects [16, 19, 23]. Furthermore, the engrossing trend was observed that the RMSE values measured by the GPS watch were increased when the wind direction was suddenly changed. However, due to the limitations of this study, not all meteorological variables were precisely monitored at the forest, and the change in wind direction was not sufficiently quantified to determine the correlation between wind direction and RMSE values.

## Conclusion

The study presented here investigated the static horizontal position accuracy of a GPS watch under varying forest cover types, seasonal fluctuations, and meteorological conditions. To evaluate the relative accuracy of the GPS watch, a mapping-grade GNSS receiver was used to compare coordinates outputs to those of the GPS watch. The key findings of this study were as follows: (1) the accuracy of the GPS watch was significantly affected by the season, but not by the cover type; (2) during the leaf-off season, the accuracy of the GPS watch did not differ significantly relative to the accuracy of the mapping-grade GNSS receiver; (3) the RMSE values of both GPS receivers had a significant but weakly positive correlation with air temperature and absolute humidity. These results suggest that canopy coverage, rather than the forest cover type, might play a more critical role in governing the static horizontal position accuracy of GPS receivers in forested areas. Furthermore, the GPS watch showed improvements in static horizontal position accuracy during the leaf-off season, so that it had a similar level of accuracy to that of a mapping-grade GNSS receiver. Our results suggest that GPS watch might be able to provide an acceptable quality of locational information for forest management purposes during the leaf-off season. However, due to the limitations inherent to a small antenna, the quality of location information might not be able to guarantee acceptable static horizontal position accuracy during the leaf-on season. A recently released GPS watch (after our data collection process was completed) that is equipped with improved GPS antenna and the Assisted-GPS (A-GPS) technology, can determine location information from network stations and various technologies employed in the mobile terminals of the Differential GPS (D-GPS). This technology uses fixed and known positions to correct the GPS signal and might provide better static horizontal position accuracy in various conditions.

Regarding the effects of meteorological variables, the GPS watch and the mapping-grade GNSS receiver both indicated a significant correlation between positional accuracy and two environmental variables (air temperature and absolute humidity). Accordingly, air temperature and absolute humidity should be considered when these types of GPS receivers are used in forested areas. Our study had some limitations in that (1) the GPS watch used for this study was released in 2014, and may therefore not be a reliable indicator of the current technological state of GPS watches; (2) the locations of nearby trees were not measured to explain the distributions of observed points, and (3) the meteorological variables within the forest were not monitored in an all-encompassing manner. Nevertheless, our study determined the accuracy of a GPS watch in various circumstances and illustrated the potential application of the GPS watch for forest management purposes, especially during the leaf-off season. Although users will need to decide whether the accuracy and reliability of a GPS watch is sufficient for their purposes, they should keep in mind that even mapping-grade GNSS receiver accuracy can vary depending on the working conditions, such as changes in canopy cover and meteorological conditions. Given falling prices and ease-of-accessibility, the GPS watch may serve as a circumstantially attractive replacement for the mapping-grade GNSS receivers. Further research exploring the technological developments in future GPS watches is therefore a relevant and useful endeavor.

## Supporting information

S1 Data Spreadsheet containing GPS data collected. (XLSX)

S2 Data Weather associated with data collection. (XLSX)

## References

[1] Gløersen ØN, Kocbach J, Gilgien M. Tracking performance in endurance racing sports: Evaluation of the accuracy offered by three commercial GNSS receivers aimed at the sports market. Frontiers in Physiology. 2018;9:1425 10.3389/fphys.2018.01425 30356794 PMC6189485

[2] Omr M, Georgy J, Noureldin A. Using multiple portable/wearable devices for enhanced misalignment estimation in portable navigation. GPS Solutions. 2017;21(2):393–404.

[3] Merry K, Bettinger P. Smartphone GPS accuracy study in an urban environment. PloS ONE. 2019;14(7). 10.1371/journal.pone.0219890 PMC6638960 31318933

[4] Bettinger P, Merry K, Bayat M, Tomaštík J. GNSS use in forestry–A multi-national survey from Iran, Slovakia and southern USA. Computers and Electronics in Agriculture. 2019;158:369–83.

[5] Danskin SD, Bettinger P, Jordan TR, Cieszewski C. A comparison of GPS performance in a southern hardwood forest: Exploring low-cost solutions for forestry applications. Southern Journal of Applied Forestry. 2009b;33(1):9–16.

[6] Fauzi M, Idris N, Yahya M, Din A, Lau A, Ishak M. Tropical forest tree positioning accuracy: A comparison of low cost GNSS-enabled devices. International Journal of Geoinformatics. 2016;12(2):59–66.

[7] Tomaštík JJ, Tomaštík JS, Saloň Š, Piroh R. Horizontal accuracy and applicability of smartphone GNSS positioning in forests. Forestry. 2016;90(2):187–98.

[8] Ordoñez C, Martínez J, de Cos Juez JF, Lasheras FS. Comparison of GPS observations made in a forestry setting using functional data analysis. International Journal of Computer Mathematics. 2012;89(3):402–8.

[9] Akbulut R, Ucar Z, Bettinger P, Merry K, Obata S. Effects of forest thinning on static horizontal positions collected with a mapping-grade GNSS receiver. Mathematical and Computational Forestry & Natural-Resource Sciences. 2017;9(1):14–21.

[10] Bettinger P, Merry K. Global Navigation Satellite System Research in Forest Management: A Summary of Horizontal, Vertical, Static, and Dynamic Accuracy Assessments: LAP Lambert Academic Publishing; 2011.

[11] Bettinger P, Fei S. One year's experience with a recreation-grade GPS receiver. Mathematical and Computational Forestry & Natural-Resource Sciences. 2010;2(2):153–60.

[12] Bettinger P, Merry K. Static horizontal positions determined with a consumer-grade GNSS receiver: One assessment of the number of fixes necessary. Croatian Journal of Forest Engineering. 2012a;33(1):149–57.

[13] Danskin S, Bettinger P, Jordan T. Multipath mitigation under forest canopies: A choke ring antenna solution. Forest Science. 2009a;55(2):109–16.

[14] Edson C, Wing MG. Tree location measurement accuracy with a mapping-grade GPS receiver under forest canopy. Forest Science. 2012;58(6):567–76.

[15] Pirti A. Using GPS near the forest and quality control. Survey Review. 2005;38(298):286–98.

[16] Ransom MD, Rhynold J, Bettinger P. Performance of mapping-grade GPS receivers in southeastern forest conditions. RURALS: Review of Undergraduate Research in Agricultural and Life Sciences. 2010;5(1):Article 2.

[17] Weaver SA, Ucar Z, Bettinger P, Merry K. How a GNSS receiver is held may affect static horizontal position accuracy. PLoS ONE. 2015;10(4):e0124696 10.1371/journal.pone.0124696 25923667 PMC4414510

[18] Bettinger P, Merry K. Influence of the juxtaposition of trees on consumer-grade GPS position quality. Mathematical and Computational Forestry & Natural-Resource Sciences. 2012b;4(2):81–91.

[19] Sigrist P, Coppin P, Hermy M. Impact of forest canopy on quality and accuracy of GPS measurements. International Journal of Remote Sensing. 1999;20(18):3595–610.

[20] Dussault C, Courtois R, Ouellet J-P, Huot J. Evaluation of GPS telemetry collar performance for habitat studies in the boreal forest. Wildlife Society Bulletin. 1999;27(4):965–72.

[21] Klimánek M. Analysis of the accuracy of GPS Trimble JUNO ST measurement in the conditions of forest canopy. Journal of Forest Science. 2010;56(2):84–91.

[22] Coster A, Williams J, Weatherwax A, Rideout W, Herne D. Accuracy of GPS total electron content: GPS receiver bias temperature dependence. Radio Science. 2013;48(2):190–6.

[23] Bevis M, Businger S, Herring TA, Rocken C, Anthes RA, Ware RH. GPS meteorology: Remote sensing of atmospheric water vapor using the Global Positioning System. Journal of Geophysical Research: Atmospheres. 1992;97(D14):15787–801.

[24] Wing MG. Consumer-grade global positioning systems (GPS) receiver performance. Journal of Forestry. 2008;106(4):185–90.

[25] Wing MG, Eklund A, Kellogg LD. Consumer-grade global positioning system (GPS) accuracy and reliability. Journal of Forestry. 2005;103(4):169–73.

[26] Yuill RS. The standard deviational ellipse; an updated tool for spatial description. Geografiska Annaler: Series B, Human Geography. 1971;53(1):28–39.

